# αβ-Dehydrocurvularin isolated from the fungus *Aspergillus welwitschiae* effectively inhibited the behaviour and development of the root-knot nematode *Meloidogyne graminicola* in rice roots

**DOI:** 10.1186/s12866-020-01738-2

**Published:** 2020-03-04

**Authors:** Chao Xiang, Ying Liu, Shi-Ming Liu, Ya-Fei Huang, Ling-An Kong, Huan Peng, Mao-Yan Liu, Jing Liu, De-Liang Peng, Wen-Kun Huang

**Affiliations:** 1 State Key Laboratory for Biology of Plant Diseases and Insect Pests, Institute of Plant Protection, Chinese Academy of Agricultural Sciences, Beijing, 100193 People’s Republic of China; 2 Bureau of Water Resource in Xingtang County, Shijiazhuang, 050600 People’s Republic of China; 3 College of Chemical Engineering, Beijing University of Chemical Technology, Beijing, 100029 People’s Republic of China; 4 College of Plant Protection, Hunan Agricultural University, Changsha, 410128 People’s Republic of China

**Keywords:** *Meloidogyne graminicola*, αβ-dehydrocurvularin, Nematicidal activity, Attractiveness, Development, Behaviour

## Abstract

**Background:**

The root-knot nematode *Meloidogyne graminicola* has become a serious threat to rice production as a result of the cultivation changes from transplanting to direct seeding. The nematicidal activity of *Aspergillus welwitschiae* have been investigated in vitro, and the disease control efficacy of the active compound has been evaluated under greenhouse and field conditions.

**Results:**

The active compound αβ-dehydrocurvularin (αβ-DC), isolated by nematicidal assay-directed fractionation, showed significant nematicidal activity against *M. graminicola*, with a median lethal concentration (LC_50_) value of 122.2 μg mL^− 1^. αβ-DC effectively decreased the attraction of rice roots to nematodes and the infection of nematodes and also suppressed the development of nematodes under greenhouse conditions. Moreover, αβ-DC efficiently reduced the root gall index under field conditions.

**Conclusions:**

To our knowledge, this is the first report to describe the nematicidal activity of αβ-DC against *M. graminicola*. The results obtained under greenhouse and field conditions provide a basis for developing commercial formulations from αβ-DC to control *M. graminicola* in the future.

## Background

The root-knot nematode (RKN) *Meloidogyne graminicola* is considered one of the most devastating pests on rice, especially in southern Africa, America and Southeast Asia [12, 19, 24]. This nematode penetrates rice roots and induces the formation of giant cells as the nutrition resource throughout its life cycle, with a typical symptom of hook-like galls on the roots [20]. *M. graminicola* impedes the uptake of water and nutrients due to the formation of giant cells in rice roots and causes significant yield losses in most rice-producing areas. The annual yield loss caused by *M. graminicola* is estimated at 16%~ 87% [8, 29, 32, 38]. *M. graminicola* is difficult to control because it has a short generation and high reproduction rate [11].

In the past few decades, chemical control (e.g., organophosphates, carbamates and chlorpyriphos) was the primary means for managing this nematode [15]. Soil application of phorate and fosthiazate before seeding and soaking rice seedlings in carbosulfan and chlorpyriphos solutions both significantly reduced root galling on rice plants [33]. However, due to the side effects of these chemicals on the environment and humans, many chemical nematicides have been withdrawn or restricted [3, 6]. Methyl bromide was completely phased out in 2015 because of its negative effect on stratospheric ozone [1]. Sulfuryl fluoride might play an important role in global warming because of its chemical stability [39]. In addition, policies have been designed to support environmentally friendly farming practices in many countries, and eco-friendly alternatives are increasingly demanded. Thus, much research has aimed to identify nematicidal metabolites from antagonistic microorganisms to control RKNs [10, 16, 35]. However, few commercial products have been developed and applied in agriculture. Therefore, the identification of antagonists is necessary for the control of RKN and its commercialization.

Biological control of endophytic fungi has been demonstrated to be effective in reducing the penetration and reproduction of plant-parasitic nematodes [21, 22, 25, 40]. Le et al. [21, 22] reported that the root-galling severity of *M. graminicola* was reduced by 38% with treatment of *Trichoderma* spp. and that *M. graminicola* penetration was significantly reduced by 55% by endophytic *Fusarium moniliforme* colonization. *Aspergillus* species are very common in soils, and many scientific studies have indicated that metabolites isolated from *Aspergillus* spp. have high potential for plant-parasitic nematode management [2]. Li et al. [23]. found that *A. niger* could reduce *M. incognita* populations and promote tomato growth. According to Jin et al. [13], metabolites of *A. niger* caused 90% mortality of second-stage juveniles and remarkably reduced the penetration of *Heterodera glycines* in soybean roots. αβ-Dehydrocurvularin (αβ-DC) was first identified as a metabolite of *A. aureofulgens* by Caputo and Viola [4]. Previous studies showed that αβ-DC isolated from different microorganisms had various antimicrobial activities and nematicidal activities [4, 18]. αβ-DC also showed weak activity against gram-positive and gram-negative bacteria [4], while βγ-DC could cause 35% mortality in *Pratylenchus penetrans* at a concentration of 300 μg mL^− 1^ [18]. However, the nematicidal activity and the action mode of αβ-DC against RKN *M. graminicola* have not yet been determined.

Therefore, further insights into the nematicidal activity of αβ-DC against *M. graminicola* were obtained in this study, which aimed to identify a potential alternative to control *M. graminicola*. To provide further evidence of the effects of αβ-DC on *M. graminicola* and to elucidate the mechanism of action, the effects of αβ-DC on nematode survival, giant cell development, locomotion behaviour and reproductive activity were evaluated at room temperature, and the infectivity of nematodes to rice plants was also evaluated under field conditions in this study.

## Results

### Identification of αβ-dehydrocurvularin from *A. welwitschiae*

**Fig. 1:**
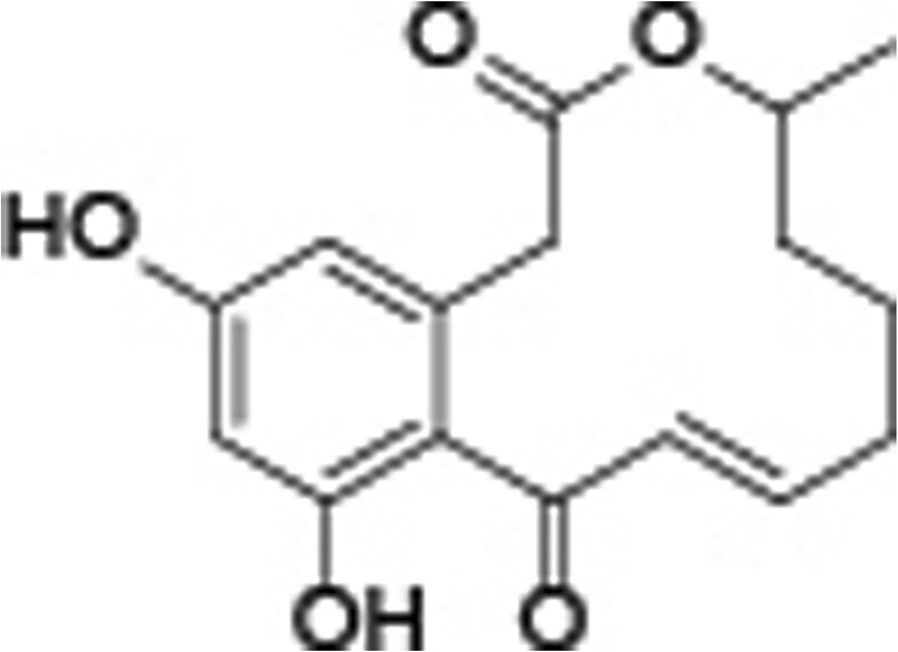
Chemical structure of αβ-dehydrocurvularin isolated from *Aspergillus welwitschiae* AW2017

In total, 15.2 g crude filtrates were extracted by CHCl_3_/MeOH eluent from fermentations of *A. welwitschiae* and were purified by column chromatography on silica gel. Then, 285 mg of the active fraction was repeatedly crystallized from Me_2_CO/EtoAc and produced as a white powder. By comparing the physiochemical properties with those reported previously, the major nematicidal compounds from *A. welwitschiae* were identified as αβ-dehydrocurvularin (αβ-DC), βγ-dehydrocurvularin and 7-oxocurvularin. Among three purified compounds, αβ-DC showed the best nematicidal activity based on a preliminary bioassay (data not shown) and was synthesized for next experiments. The chemical structure of αβ-DC was shown in Fig. 1.

### Nematicidal effects of αβ-DC on *M. graminicola* juveniles

**Fig. 2:**
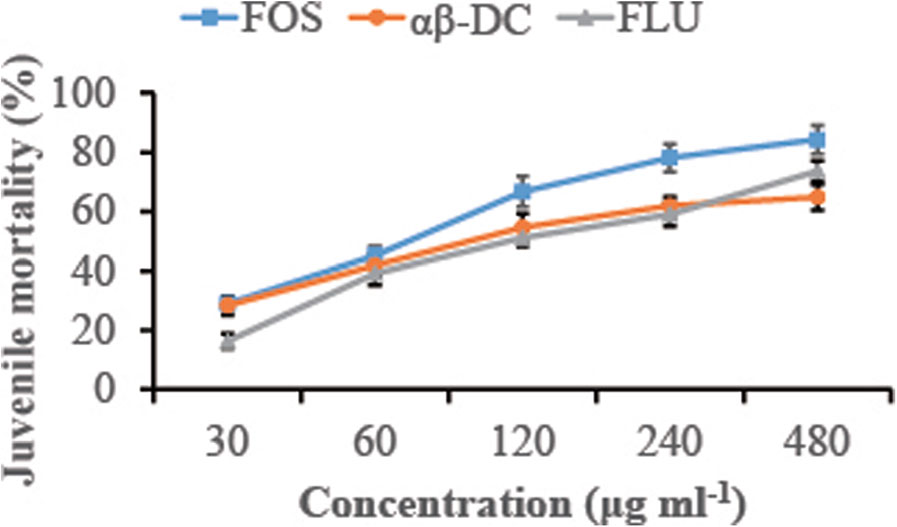
Concentration-mortality curves of *Meloidogyne graminicola* exposed to αβ-DC, fosthiazate or fluopyram for 48 h. Values are the mean ± SE of six replicates from three trials

To identify the nematicidal effect of αβ-DC on the *M. graminicola* juveniles, LC_50_ of αβ-DC on this nematode was analyzed and compared with two formulated nematicides. The mortality curves of *M. graminiicola* exposed to fosthiazate (FOS), αβ-DC and fluopyram (FLU) were investigated (Fig. 2). The LC_50_ values of FOS, αβ-DC and FLU were 89.1, 122.2 and 139.6 μg mL^− 1^, respectively. It was remarkable that FOS displayed higher nematicidal activity than αβ-DC and FLU (*P* ≤ 0.05), although at the lower concentration (≤120 μg mL^− 1^) no difference could be observed between these compounds. No significant difference was observed between αβ-DC and FLU, except at the concentration of 30 μg mL^− 1^. These data suggested that the nematicidal activity of αβ-DC was similar to that of FLU but was significantly lower than that of FOS at tested concentrations.

### Effect of αβ-DC on the attractiveness of rice roots to *M. graminicola* and giant cell development

**Fig. 3:**
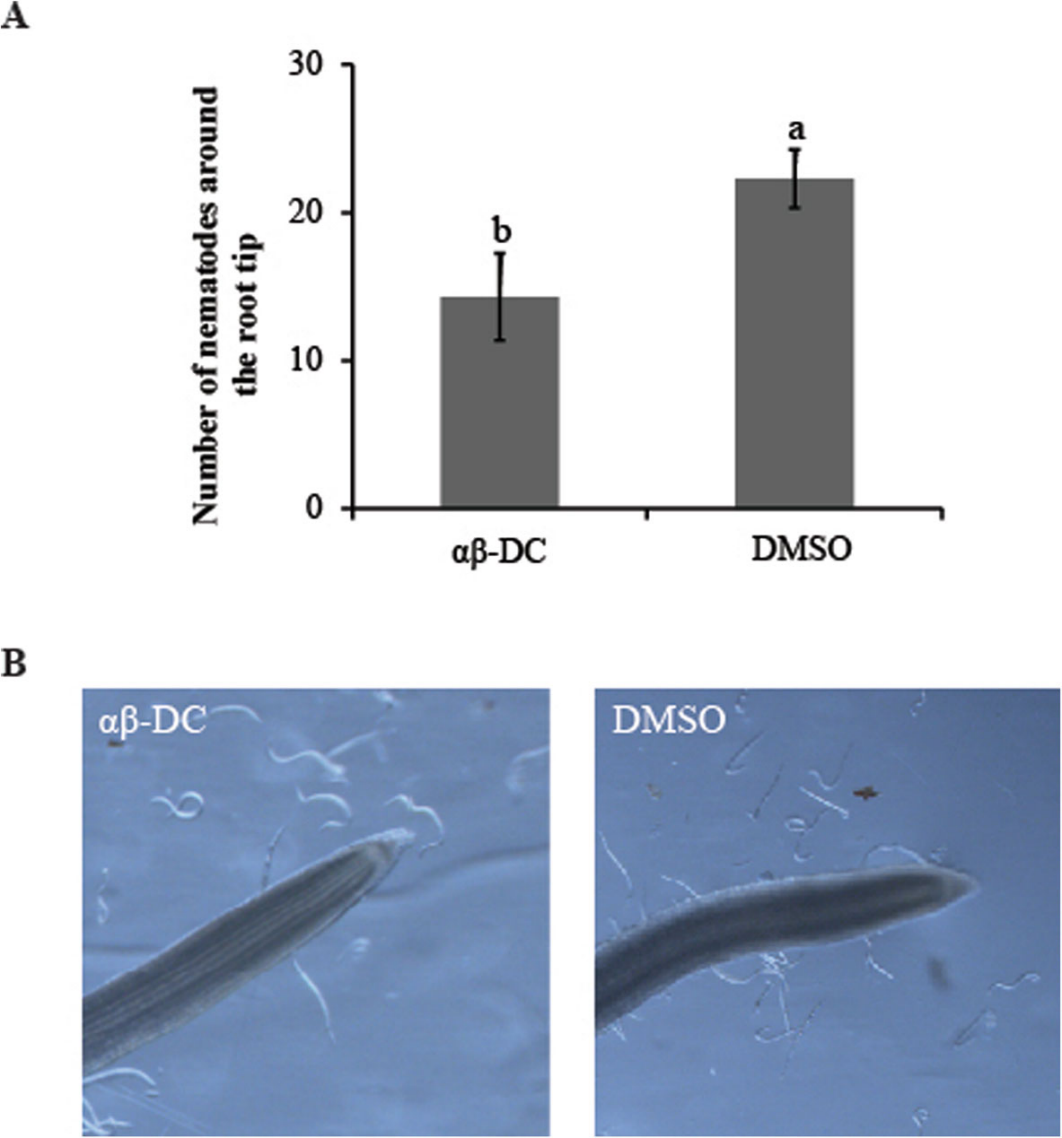
Attraction of *M. graminicola* towards the rice root tip after drenching with αβ-DC or DMSO solution. **a**. Nematodes attracted to within 5 mm around the root tip were counted at 6 hpi. **b**. Nematodes around the root tip were photographed under a Leica stereomicroscope with a DFC400 camera. The entire experiment was performed thrice, with four replicates

To determine whether αβ-DC has a direct effect on the attractiveness of roots to *M. graminicola*, we counted the numbers of nematodes attracted to within 5 mm around the roots treated with αβ-DC or DMSO solution. At 6 h post inoculation (hpi), the numbers of nematodes attracted to the αβ-DC-treated root tips (14.3 ± 2.9) were significantly lower than those attracted to the control roots (22.3 ± 2.0) (*P* ≤ 0.05)(Fig. 3a and b). This result indicates that the tested chemicals prevented the attraction of rice roots to *M. graminicola*.

**Fig. 4:**
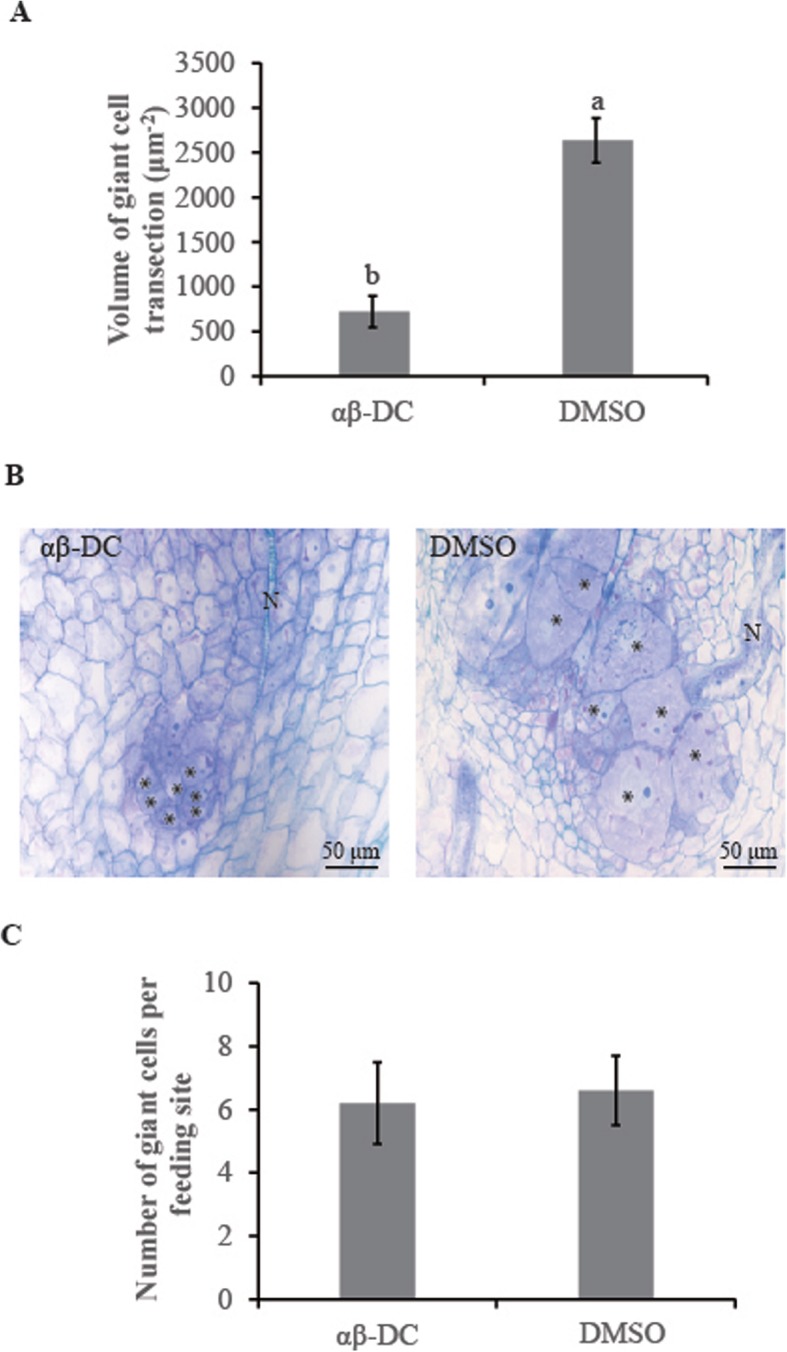
Analysis of giant cell structures in rice root galls after treatment with αβ-DC or DMSO solution. **a** Volume of each giant cell transection. **b**. Giant cells (*) induced by the nematode (N) in αβ-DC-amended root galls and DMSO solution were stained at 7 dpi with toluidine blue solution and photographed under an Olympus DP74 (bar = 50 μm). N denotes the nematode. **c**. Numbers of giant cells at each feeding site. The experiment was repeated twice, with ten galls in each treatment

A microscopic investigation of giant cells revealed a significant difference in the morphological characteristics of feeding sites in the αβ-DC-treated roots versus the non-treated roots. The volume of giant cell transections in the αβ-DC-treated roots (720.3 ± 176.2 μm^2^) was significantly smaller than that in the DMSO-treated roots (2636.1 ± 250.2 μm^2^) (*P* ≤ 0.05) (Fig. 4a and b). However, no significant differences were observed in the numbers of giant cells per feeding site between the αβ-DC-treatment (6.2 ± 1.3) and DMSO-treated control (6.6 ± 1.1) (Fig. 4c). These data demonstrate that the αβ-DC treatment had a negative effect on giant cell development, thus decreasing the supply of nutrients to the nematodes.

### Effect of αβ-DC on the development and infectivity of nematodes

**Fig. 5:**
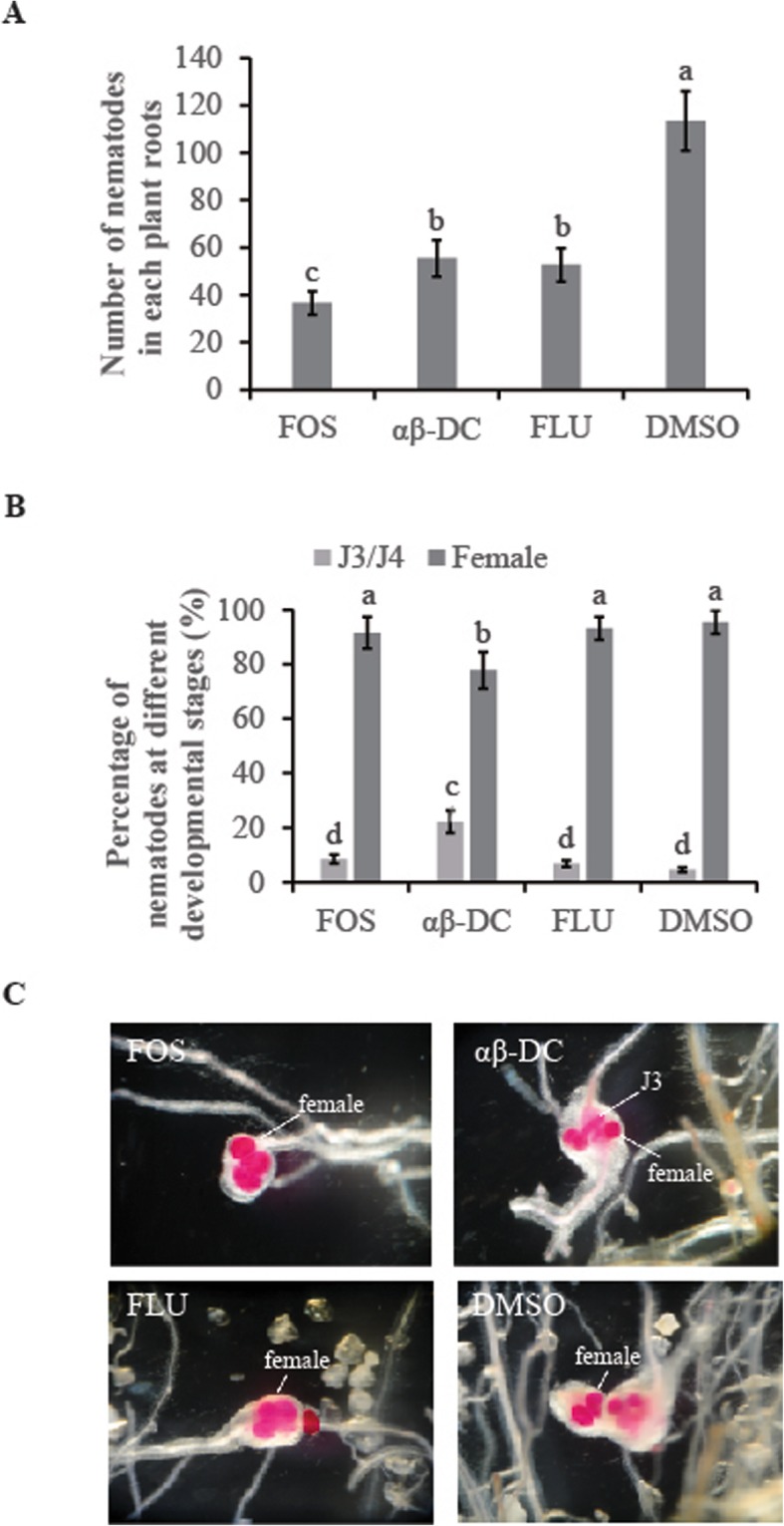
Effect of αβ-DC amendment on nematode infection and development under greenhouse conditions. **a**. Nematodes in each αβ-DC, fosthiazate or fluopyram-amended root system were counted at 14 dpi. Drenching with DMSO solution was used as a control. **b**. The ratio of nematodes in rice roots at different developmental stages is the value of the numbers of nematodes in different life stages (female or J3/J4) divided by the total numbers of nematodes in roots. **c**. Nematodes in root galls were stained with acid fuchsin and photographed under a Leica stereomicroscope with a DFC400 camera. The bars in the different graphs represent the mean ± SE of data from three independent biological replicates, each containing 6 individual plants. Different letters indicate statistically significant differences (Duncan’s multiple range test at *P* ≤ 0.05)

To evaluate the effect of αβ-DC on *M. graminicola* infectivity and development, nematodes in rice plants drenched with αβ-DC, FOS, FLU or DMSO solution, respectively, were counted in a greenhouse experiment. Drenching with αβ-DC significantly decreased nematode penetration compared with control plants. At 14 day post inoculation (dpi), the observation of nematodes in fuchsin-stained roots showed that the nematode numbers in the FOS treatment (36.6 ± 4.9) were significantly lower than those in the αβ-DC treatment (55.4 ± 7.7) and FLU treatment (52.7 ± 7.0), and all the treatments resulted in a significant reduction in total nematode numbers compared to the control roots (113.4 ± 12.5) (*P* ≤ 0.05) (Fig. 5a). These data suggest that drenching with αβ-DC decreased nematode infection in rice roots.

According to their normally developmental progression, most J2s of *M. graminicola* will develop into female-stage at 14 dpi. Thus, the percentage of female-stage nematodes among observed nematodes in rice roots will be used to assess whether nematode development is delayed or not. As shown in Fig. 5b and c, the percentage of females in αβ-DC-treated roots (77.8 ± 6.7%) was significantly lower than that in control roots (95.4 ± 4.3%) (*P* ≤ 0.05), where a higher ratio of J3/J4s was observed in αβ-DC-treated plants (22.2 ± 4.2%) than that in control plants (4.6 ± 0.9%). These results implied that the development of nematodes in αβ-DC-treated plants was slightly delayed compared with the control plants. However, no significant difference in the percentage of females was observed among the control plants, FOS-treated plants and FLU-treated plants. These data suggest that drenching with αβ-DC delayed nematode development inside the rice roots.

**Fig. 6:**
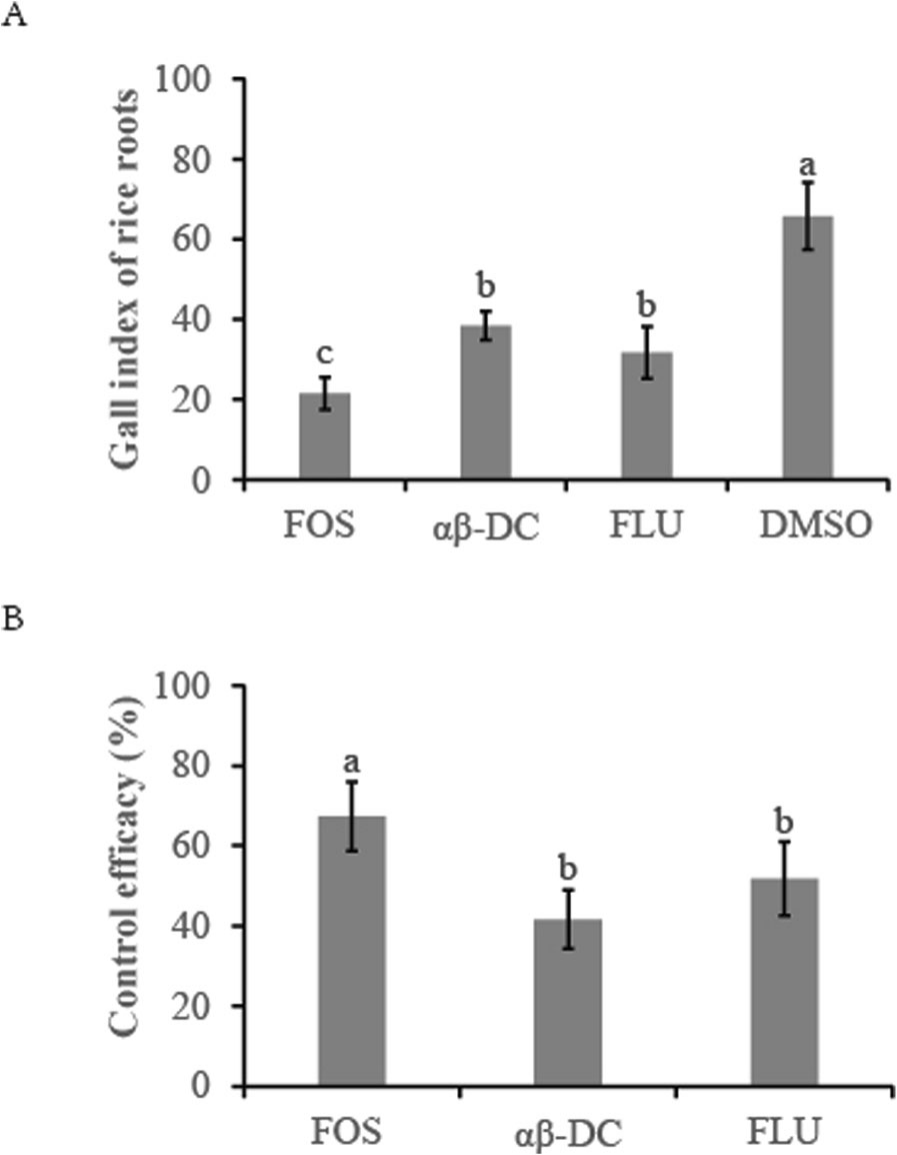
Effect of αβ-DC amendment on nematode infection under field conditions. **a**. Root gall index in αβ-DC, fosthiazate or fluopyram-amended root systems was statistically analysed at 50 d after direct seeding. Twenty seedlings from each plot were uprooted and analysed. **b**. The control efficacy in different treatments. Treatments in both 2017 and 2018 were evaluated in a randomized block design with 4 replicates. Different letters indicate statistically significant differences (Duncan’s multiple range test at *P* ≤ 0.05)

Root galls are typically a direct reflection of nematode infection levels and always correlate with the susceptibility of rice varieties to nematodes [42]. To evaluate the effect of αβ-DC on the infection of nematodes under field conditions, a field experiment was conducted on a commercial rice base that had been naturally infested with *M. graminicola* for more than 10 years. Field experiments also demonstrated the negative effect of αβ-DC on the infectivity of *M. graminicola*. At 50 d after direct seeding, the root gall index of rice roots was significantly decreased in all the treatments (Fig. 6a). The lowest root gall index was observed in the FOS treatment (21.5 ± 4.1), which significantly differed from the FLU treatment (31.7 ± 6.5) and the αβ-DC treatment (38.4 ± 3.6). No significant differences in the root gall index were observed between αβ-DC and FLU treatments. The highest root gall index was observed in the DMSO control (65.8 ± 8.4). Among the three chemical treatments, the FOS treatment exhibited the highest control efficacy (67.3 ± 8.6%) in reducing the root gall index, followed by the FLU treatment (51.8 ± 9.5%), while the αβ-DC treatment showed the lowest control efficacy (41.6 ± 7.4) (Fig. 6b). These data suggest that drenching with αβ-DC markedly decreased the infection of *M. graminicola* in rice fields.

## Discussion

The root-knot nematode *M. graminicola* has been considered to be a serious threat to rice production with the transformation of cultivation modes from transplanting to direct seeding. Consequently, new biologically active metabolites are required to qualify the increasing demand for controlling *M. graminicola*. In the present study, we demonstrated that αβ-DC extracted from *A. welwitschiae* was toxic to *M. graminicola* juveniles. The application of αβ-DC decreased the attractiveness of rice roots to nematodes, reduced nematode infection and retarded the development of nematodes in rice roots. These results indicate that the natural product αβ-DC isolated from *A. welwitschiae* may be a highly promising source of chemicals to manage *M. graminicola*. To our knowledge, this is the first report to detail the suppression effect of αβ-DC on *M. graminicola*.

After measurement of the toxic effect of this compound on nematodes, further research was conducted to determine the action mode of αβ-DC, with analyses of nematode infectivity, development and attraction behaviour after application of αβ-DC. To date, only a few host- or non-host-specific compounds are known to mediate the attractiveness of crops to nematodes [7, 22, 28]. Diez and Dusenbery [7] observed that carbon dioxide or amino acids helped RKN *M. incognita* to trace tomato roots. In our previous research, we found that αβ-DC might inhibit the motility of the nematode in a chamber experiment [24]. The inoculation of 2-week-old seedlings with *A. welwitschiae* filtrates reduced the attractiveness of rice roots to *M. graminicola*, resulting in *ca.* 40% reduction in juveniles migrating to 1.5–4.5 cm from rice roots. Based on the present research, it is evident that the application of αβ-DC significantly decreased the attractiveness of rice roots to *M. graminicola*, thus hindered nematode invasion and subsequent infection. Le et al. [22] observed that the root exudates from *F. moniliforme* Fe14-treated plants decreased the attractiveness of *M. graminicola* to host roots. However, root exudates from *Pseudomonas fluorescens*-treated plants had no effects on the attractiveness of cyst nematode *H. schachtii* in sugar beet [28]. Thereafter, the principle method by which nematodes locate different host roots remains to be discovered.

As previously reported, dehydrocurvularin is a unique compound produced by *Aspergillus* spp. and has various antimicrobial activities and nematicidal activities [4, 18, 34]. Kusano et al. [18] reported that βγ-DC caused a mortality of 35% in *Pratylenchus* spp. at a concentration of 300 μg mL^− 1^. In the present study, our experimental results indicated that αβ-DC had significant nematicidal activity against *M. graminicola* in an in vitro test, with an LC_50_ of approximately 122.2 μg mL^− 1^, which is similar to the effect of the chemical nematicide fluopyrum. However, the nematicidal activity of αβ-DC against J2s of *M. graminicola* was significantly higher than that of βγ-DC against *P. penetrans*. These results strongly suggested that the nematicidal activity of αβ-DC was closely related to its strong arrest of infection. Therefore, αβ-DC can be used as a promising biopesticide for the control of RKN.

The slowing reproduction of *M. graminicola* in the greenhouse experiment also indicated that αβ-DC retarded nematode development in rice roots. In previous investigations, some chemicals have been demonstrated to arrest larval development [30]. Exogenous chemicals (e.g., benomyl, nocodazole) or starvation affected cellular processes in the embryo, larvae or adult germline of *Caenorhabditis elegans*. Furthermore, Kearn et al. [14] found that fluensulfone suppressed the development of *C. elegans* by interfering with larval moulting. However, the mechanism underlying the arrested development caused by αβ-DC remains unclear. In the present research, the volume of giant cell transections in the αβ-DC-treated roots was significantly lower than that in the non-treated roots. Based on the morphological changes of giant cells, we speculated that drenching with αβ-DC retarded the development of giant cells, which might decrease the food supply of nematodes and thus suppress the growth, development and cell cycle progression of *M. graminicola*. However, the exact action mechanism of αβ-DC against nematode development remains to be verified in the future.

With the banned use of many chemical nematicides, searches for biological control agents and more environmentally benign control methods are ongoing [36, 37]. In the present field experiment, the drenching of αβ-DC significantly decreased the root gall index of rice roots, with the control efficacy as high as 40% in controlling *M. graminicola*, which was similar to the effect of chemical fluopyrum. This suggests that αβ-DC can remarkably decrease the infection of *M. graminicola* in rice fields.

Based on the results of this study, αβ-DC exhibited significant nematicidal activity against *M. graminicola* and provided substantial control of RKN in the field, which implies that αβ-DC will be a promising control agent for *M. graminicola*. The mode of its action was also elucidated with analyses of nematode infectivity, development and attraction behaviour. However, further research is required to determine its effect on crops and the environment and to then develop commercial formulations with high efficacy and low cost in farm practices.

## Conclusion

To our knowledge, this is the first report to describe the nematicidal activity of αβ-DC against *M. graminicola*. αβ-DC effectively decreased the infection of nematodes, and suppressed the behaviour and development of nematodes in rice roots, which provide a basis for developing commercial formulations from αβ-DC to control *M. graminicola* in the future.

## Methods

### Identification of nematicidal compounds from *A. welwitschiae*

The *A. welwitschiae* strain AW2017 was cultured on potato dextrose broth (PDB) medium (50 L) at 25 °C for 5 d [24]. The nematicidal compounds were isolated from AW2017 metabolites as described by Kusano et al. [18] with minor modifications. Briefly, the culture filtrate was extracted with CHCl_3_/MeOH eluent and evaporated under reduced pressure. The crude material was purified by column chromatography on silica gel, and the active fractions were repeatedly crystallized from Me_2_CO/EtoAc to produce nematicidal compounds, which were used to analyse its nematicidal activities simultaneously. Final products were identified by comparing the physiochemical properties with those reported previously [4, 18, 34]. All the chemicals used in the present research were purchased from Sigma-Aldrich (China).

### Rice culture and propagation of the nematode

Rice seeds (*Oryza sativa* cv. Nipponbare) were originally obtained from the US Department of Agriculture (GSOR-100) and multiplied in Hunan Province, China. Seeds were soaked in 5.25% sodium hypochlorite for 5 min and germinated at room temperature (25 ± 4 °C) for 4 d. One geminated seed was sown in a polyvinylchloride (PVC) tube containing synthetic absorbent polymer (SAP) [43]. Rice seedlings grew in a greenhouse and were irrigated with 20 ml of Hoagland’s solution twice per week at 28 ± 2 °C with 70–75% relative humidity.

The *M. graminicola* population was propagated on the susceptible rice variety Nipponbare at 28 ± 2 °C. Nematode eggs were separated from the root galls and hatched in a 75-μm sieve at room temperature for 3–5 d. Hatched second-stage juvenile (J2) suspensions were filtered through a 25-μm sieve and resuspended in distilled water at approximately 200 nematodes mL^− 1^ for the subsequent experiments [27, 43].

### Nematicidal activity of αβ-DC against *M. graminicola* juveniles

To test the larvicidal activity of αβ-DC, approximately 200 hatched J2 individuals were added to each well of 24-well plates containing 1 ml of αβ-DC solution at 480, 240, 120, 60, and 30 μg mL^− 1^. Formulated fosthiazate emulsifiable concentrate (486 mg mL^− 1^ EC) and fluopyram suspension concentrate (417 mg mL^− 1^ SC) were also diluted to 480, 240, 120, 60, 30 μg mL^− 1^ as described above and used as positive controls. A treatment with only l ml of a 1% dimethyl sulfoxide (DMSO) water solution was included as the negative control. Four replicates were set for each treatment. The plates were kept at 25 °C for 48 h. Nematodes were considered dead if their bodies were motionless and straightened after stimulation with 1 M NaOH [5]. The dead and alive juveniles were counted under a stereomicroscope, respectively, and the corrected mortality values of juveniles were calculated as described in Liu et al. [24]. The median lethal concentration (LC_50_) of αβ-DC and other chemicals against nematodes were analysed with SAS software (SAS Institute, Cary, NC). Three trials with four replicates were performed for this experiment.

### Effect of αβ-DC on the attractiveness of rice roots to *M. graminicola*

As described in Wang et al. [41], 23 g pluronic F-127 powder was fully dissolved in 100 mL of sterile water at 4 °C while stirring for 24 h. Then, an attraction bioassay was performed at room temperature. Rice roots of 2-week-old plants were drenched with 20 mL αβ-DC (30 μg mL^− 1^) solution or 1% DMSO water solution. One day later, a 1-cm-long root tip was cut and placed into a six-well culture plate containing 1 mL pluronic F-127 gel and approximately 100 hatched J2s. Nematodes attracted to 5 mm around the root tip were counted under a Leica stereomicroscope and were photographed with a DFC400 camera at 6 hpi. The entire experiment was performed thrice, with four replicates.

### Morphological observation of nematode giant cells

After J2s enter the vascular cylinder, they inject pharyngeal secretions to induce permanent feeding sites known as giant cells, which are used as food resources throughout their life cycle [9]. To observe the morphological changes of nematode giant cells, each rice plant was drenched with 20 ml αβ-DC (30 μg mL^− 1^). Plants drenched with DMSO solution were used as the control. One day later, each plant was inoculated with 100 J2 s. At 7 dpi, 10 root galls from six plants were fixed in 1 × PIPES buffer overnight and then dehydrated in different ethanol dilutions. After being embedded in Technovit 7100 for 2 weeks, gall tissues were sectioned into 10-μm slices with a Leica RM2265 (Leica Microsystems, Beijing, China). Slices on glass slides were stained with 0.05% toluidine blue and sealed with DPX mountant. Microscopic observations were performed under an Olympus SZX 16 at 40× magnification, and images were obtained with an Olympus DP74 (Olympus Optical Company, Tokyo, Japan). The experiment was repeated twice.

### Development of nematodes in greenhouse experiments

To determine the direct effect of αβ-DC on the development of nematodes, each rice roots was drenched with 20 mL αβ-DC (240 μg mL^− 1^), fosthiazate EC (48.6 μg mL^− 1^), fluopyram SC (4.17 μg mL^− 1^) or 1% DMSO solution, respectively, at 24 h before nematode inoculation. Each 2-week-old rice plant was inoculated with 200 J2s around the roots and maintained in the greenhouse at 28 ± 2 °C. At 14 dpi, root samples were collected, and nematodes in rice roots were counted under a stereomicroscope after staining with acid fuchsin for 3 min [26]. The total numbers of nematodes in the third or fourth stage (J3/J4) and females were counted. To calculate the ratio of nematodes at various stages, the numbers of nematodes in different life stages (female or J3/J4) were divided by the total numbers of nematodes in roots using Microsoft Excel 6.0 (Redmond, Washington, USA). The entire experiment was performed thrice, each containing 6 individual plants.

### Infectivity of nematodes in the field experiment

The field experiment was continuously carried out in a commercial direct-seeding field in Hunan Province, China, from July to September in 2017 and 2018. Rice had been cultivated in this field for at least 40 years and was naturally infested with *M. graminicola* for 10 years [43]. To ensure the nematodes were evenly distributed in the field, the soil was mixed well with a rotary cultivator. To prevent the spread of nematodes, each plot (4 m × 5 m) was constructed with an earthen levee (height of 25 cm and width of 30 cm) as described in Khanam et al. [17]. In total, four treatments were evaluated in a randomized block design, with 4 replicates, as follows: (1) 480 mg mL^− 1^ αβ-DC (15 ml m^− 2^), (2) 486 mg mL^− 1^ fosthiazate EC (2 ml m^− 2^), (3) 417 mg mL^− 1^ fluopyram SC (0.15 ml m^− 2^), and (4) untreated control with only 2 L 1% DMSO solution. Each chemical agent was diluted with 2 L DMSO solution and sprayed evenly on the nursery bed before seeding. Approximately 400 g of seeds were directly seeded on each plot after soaking in water at room temperature for 9 h. At 50 d after direct seeding, 20 seedlings from each plot were uprooted and washed free of soil. Root galling was rated on a scale of 0 to 5, where level 0 = no galls, level 1 = 1~2, level 2 = 3~10, level 3 = 11~20, level 4 = 21~30, and level 5 ≥ 30 galls per root system [31]. The gall index was calculated using the formula described in Zhan et al. [43]. The control efficacy was calculated according to the following formula: control efficacy (%) =100× (gall index of control-gall index of treatment) /gall index of control.

### Statistical analysis

The mean and standard errors (SE) of the data were subjected to statistical analysis using SAS software version 8.0 (SAS Institute, Cary, NC). Significant differences (*P* ≤ 0.05) between the treatments were determined according to Duncan’s multiple range test.

## Data Availability

The datasets used and/or analysed during the current study available from the corresponding author on reasonable request.

## Abbreviations

DMSO: Dimethyl sulfoxide
dpi: Day post inoculation
FLU: Fluopyram
FOS: Fosthiazate
hpi: Hour post inoculation
J2: Second-stage juvenile
LC_50_: Median lethal concentration
PDB: Potato dextrose broth
RKN: Root-knot nematode
SAP: Synthetic absorbent polymer
αβ-DC: αβ-dehydrocurvularin
